# Supplementary material for: Hydrodynamic Characteristics and Conformational Parameters of Ferrocene-Terpyridine-Based Polymers
Source: Polymers (Basel). 2022 Apr 27;14(9):1776. doi: 10.3390/polym14091776 (PMC9104623; doi:10.3390/polym14091776)
Supplement: Supplementary file 1 [file polymers-14-01776-s001.zip › polymers-1590206-Supplementary.pdf]

polymers

Supporting Information

Hydrodynamic Characteristics and Conformational Parameters of Ferrocene-Terpyridine Based Polymers

Alexander S. Gubarev [1], Alexey A. Lezov [1], Igor Perevyazko [1], Nina G. Mikusheva [1], Alexandra A. Lezova [1], Anna S. Senchukova [1], Anna N. Podsevalnikova [1], Vyacheslav B. Rogozhin [1], Marcel Enke [2,3], Andreas Winter [2], Ulrich S. Schubert [2,3,*] and Nikolai V. Tsvetkov [1,*]

[1] Department of Molecular Biophysics and Polymer Physics, St. Petersburg University, Universitetskaya Nab. 7/9, 199034 Saint-Petersburg, Russia; a.gubarev@spbu.ru (A.S.G.); a.a.lezov@spbu.ru (A.A.L.); i.perevyazko@spbu.ru (I.P.); n.mikusheva@spbu.ru (N.G.M.); a.lezova@spbu.ru (A.A.L.); grease_91@mail.ru (A.S.S.); a.podsevalnikova@spbu.ru (A.N.P.); v.rogozhin@spbu.ru (V.B.R.)

[2] Laboratory of Organic and Macromolecular Chemistry (IOMC), Friedrich Schiller University Jena, Humboldtstr. 10, 07743 Jena, Germany; marcel.enke@uni-jena.de (M.E.); andreas.winter@uni-jena.de (A.W.)

[3] Jena Center for Soft Matter (JCSM), Friedrich Schiller University Jena, Philosophenweg 7, 07743 Jena, Germany

* Correspondence: ulrich.schubert@uni-jena.de (U.S.S.); n.tsvetkov@spbu.ru (N.V.T.)

Table S1. The experimentally determined solvent parameters[1] used in hydrodynamic study.

	ρ_0, g/cm^3	η_0, cP
	20 °C	
THF	0.8878	0.50
THF-d8	0.9903	0.54
Toluene	0.8669	0.589
DMF	0.9490	0.82

[1] – solvent density ρ_0 and dynamic viscosity η_0.

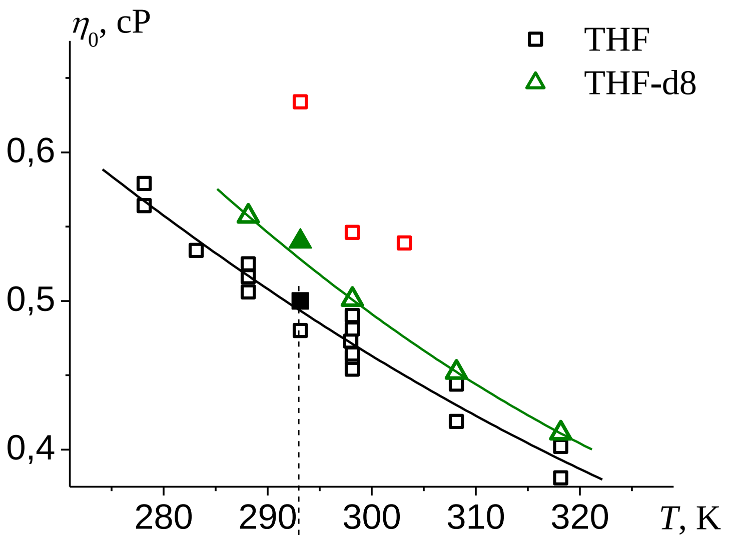

Figure S1. The dynamic viscosity data on THF (squares) and THF-d8 (triangles) known from the literature (open symbols) [1-8] and determined experimentally (filled symbols) in this study. The data points in red were not included in extrapolation of data (solid lines).

Table S2. Experimental details for the polymer synthesis.

Polymer	Synthesis details
poly(Fc)	Fc monomer: 0.750 g (2.192 mmol)<br>CPDB: 0.846 mg (9.13 × 10-3 mmol); monomer-to-CPDB ratio: 240; 0.607 mL of a stock solution (6.66 mg CPDB in 2 mL toluene)<br>AIBN: 0.375 mg (2.28 × 10-3 mmol); CPDB-to-AIBN ratio: 4; 0.347 mL of a stock solution (2.16 mg AIBN in 2 mL toluene)<br>Toluene: 1.786 mL
poly(tpy)	Tpy monomer: 0.415 g (0.994 mmol)<br>CPDB: 2.02 mg (3.82 × 10-3 mmol); monomer-to-CPDB ratio: 260; 0.447 mL of a stock solution (5.68 mg CPDB in 3 mL toluene)<br>AIBN: 0.157 mg (9.55 × 10-4 mmol); CPDB-to-AIBN ratio: 4; 0.175 mL of a stock solution (2.69 mg AIBN in 3 mL toluene)<br>Toluene: 0.620 mL
(Fc)-*co*-(tpy)	Fc monomer: 0.246 g (0.719 mmol)<br>Tpy monomer: 0.300 g (0.719 mmol)<br>CPDB: 1.325 mg (5.99 × 10-3 mmol); monomer-to-CPDB ratio: 240; 0.543 mL of a stock solution (4.88 mg CPDB in 2 mL toluene)<br>AIBN: 0.246 mg (1.49 × 10-3 mmol); CPDB-to-AIBN ratio: 4; 0.168 mL of a stock solution (2.93 mg AIBN in 2 mL toluene)<br>Toluene: 1.085 mL
(Fc)-*bl*-(tpy)	Fc monomer: 0.233 g (0.652 mmol)<br>Tpy monomer: 0.300 g (0.719 mmol)<br>poly(tpy): 67.88 mg (2.72 × 10-3 mmol); monomer-to-poly(tpy) ratio: 240<br>AIBN: 0.111 mg (6.78 × 10-4 mmol); poly(tpy)-to-AIBN ratio: 4; 0.151 mL of a stock solution (2.22 mg AIBN in 3 mL toluene)<br>Toluene: 0.664 mL

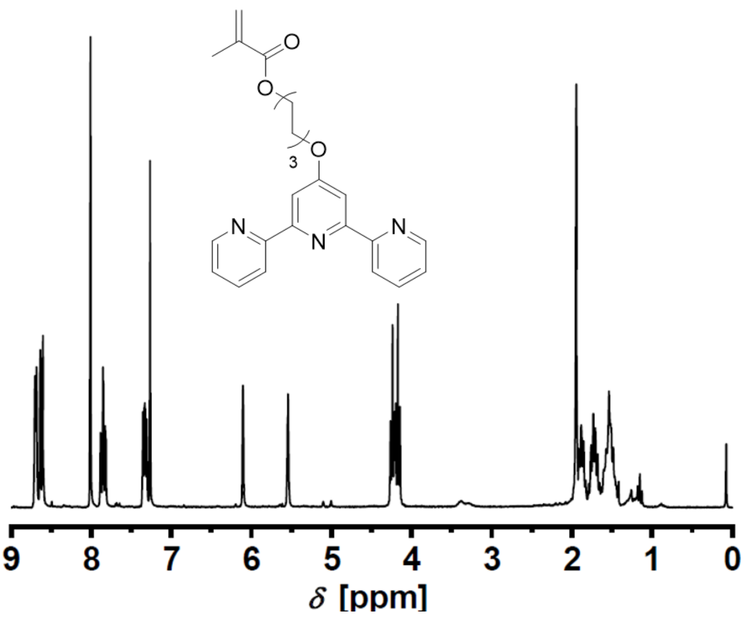

Figure S2. ^{1}H-NMR spectra of the tpy monomer (**1**, 300 MHz, 25 °C, $CDCl_3$).

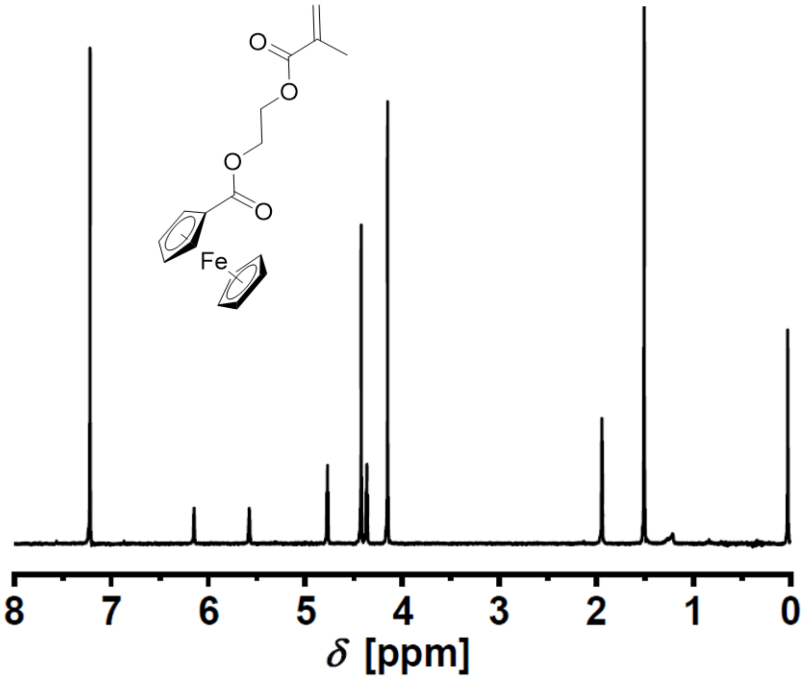

Figure S3. ^{1}H-NMR spectra of the Fc monomer (**2**, 300 MHz, 25 °C, $CDCl_3$).

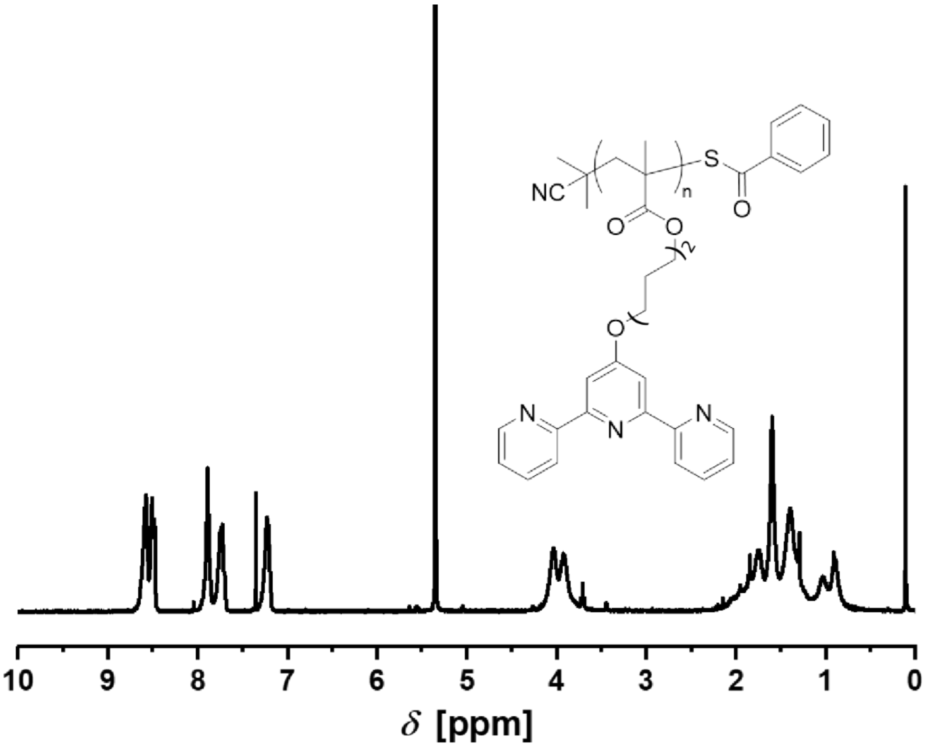

Figure S4. ^{1}H-NMR spectra of *poly*(tpy) (300 MHz, 25 °C, CD_2Cl_2).

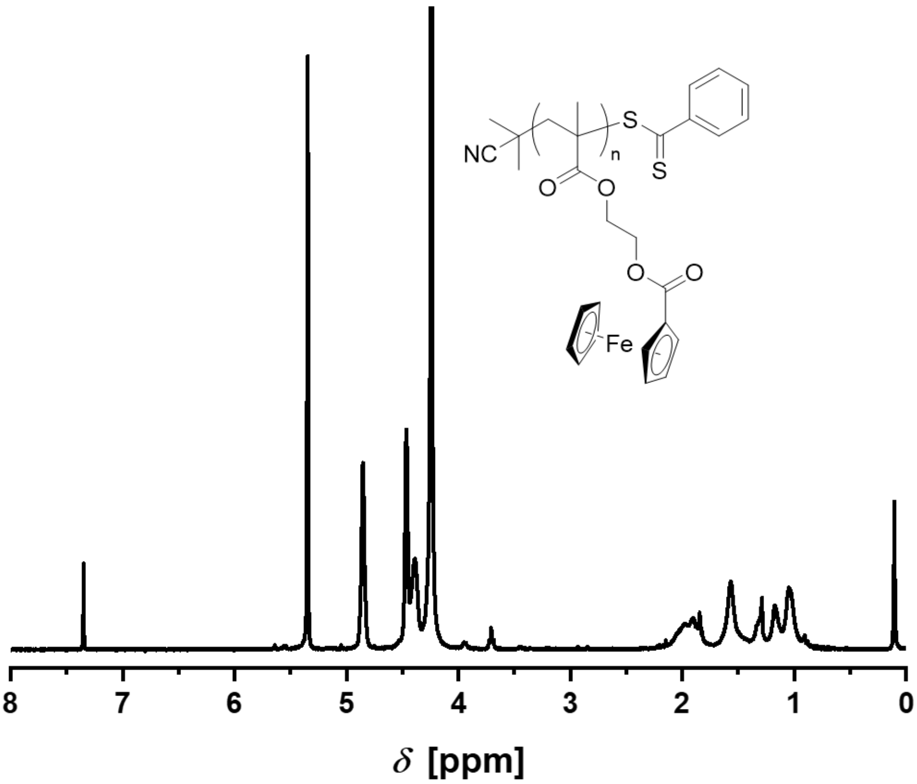

Figure S5. ^{1}H-NMR spectra of *poly*(Fc) (300 MHz, 25 °C, CD_2Cl_2).

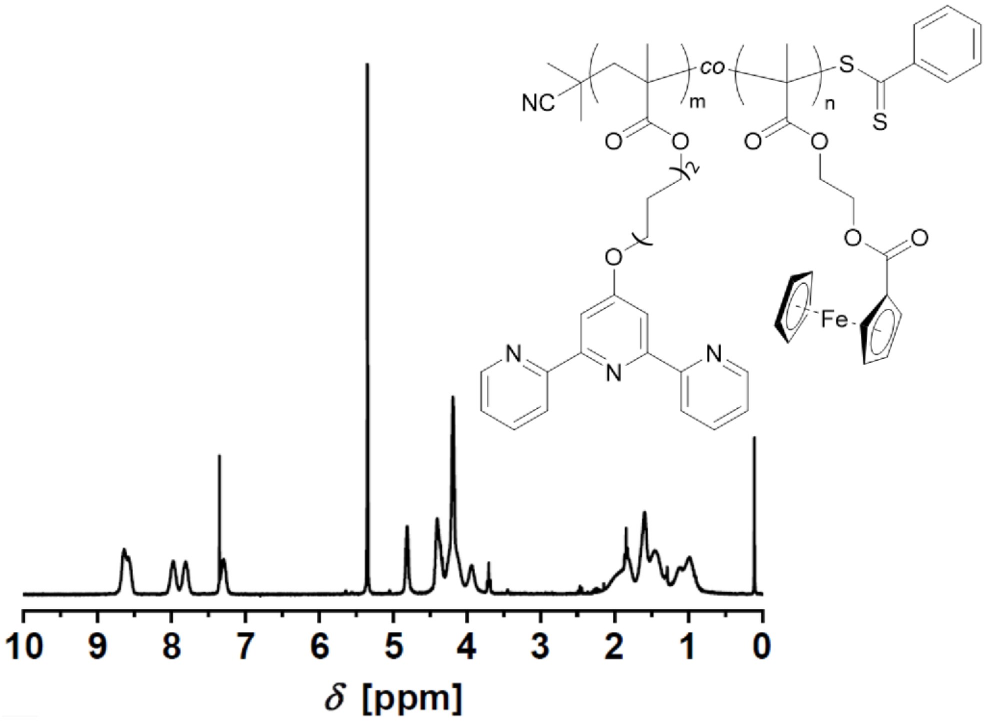

Figure S6. ^{1}H-NMR spectra of (Fc)-*co*-(tpy) (300 MHz, 25 °C, CD_2Cl_2).

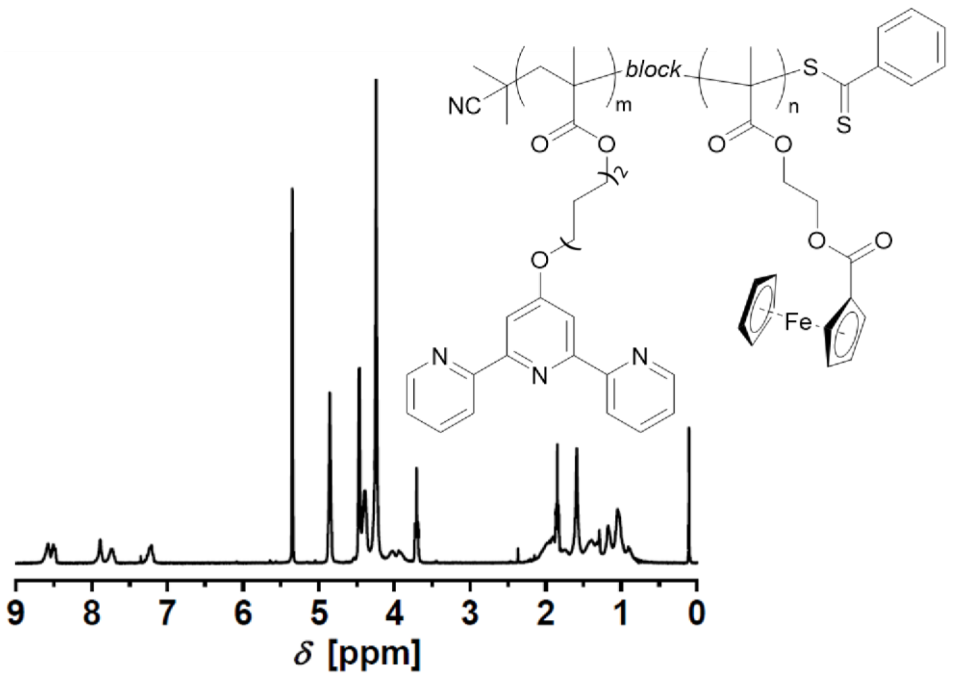

Figure S7. ^{1}H-NMR spectra of (Fc)-*bl*-(tpy) (300 MHz, 25 °C, CD_2Cl_2).

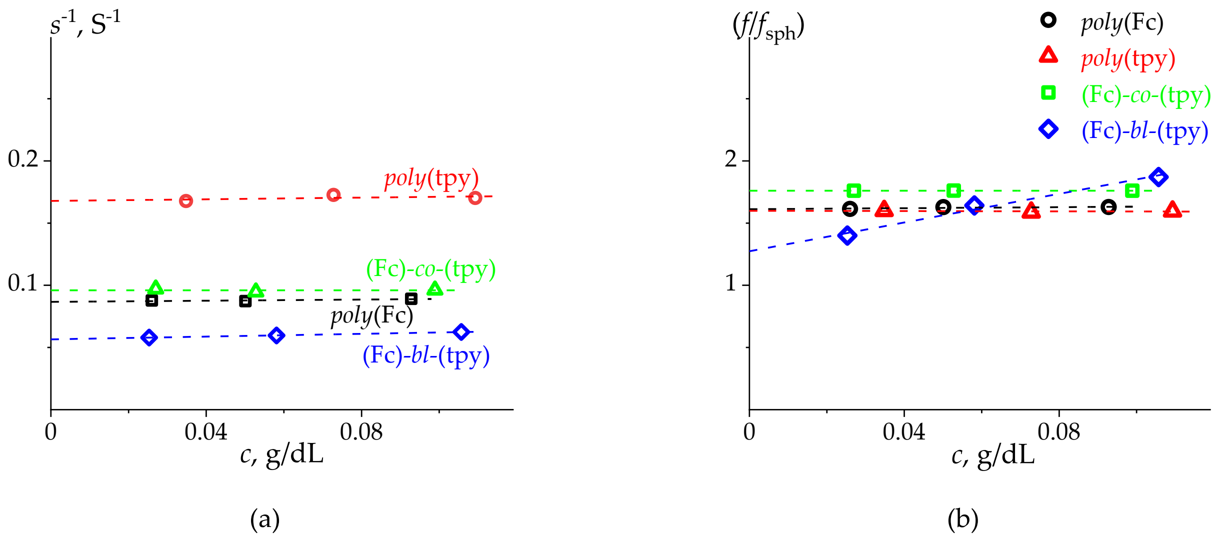

Figure S8. The concentration dependences of reciprocal sedimentation coefficients s^{-1} (a) and frictional ratios (f/f_{sph}) values (b) resolved with Sedfit for all of the studied systems in THF solutions. All data were obtained at 20 °C.

Table S3. The initial DLS data: inverse relaxation times $1/\tau$ vs. scattered vector squared q^2 acquired for the studied systems at 20 °C.

Sample	Solvent	*c*, g/dL	$1/\tau$ vs. q^2
	THF	0.61	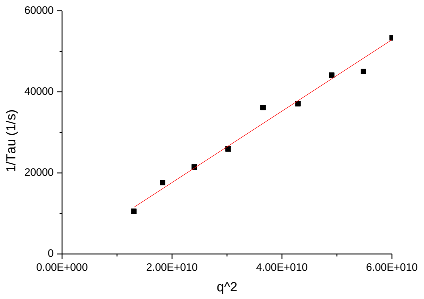
poly(Fc)	Toluene	0.71	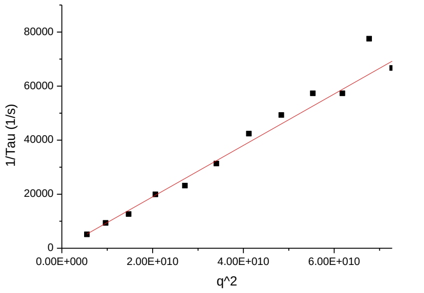
	DMF	0.13	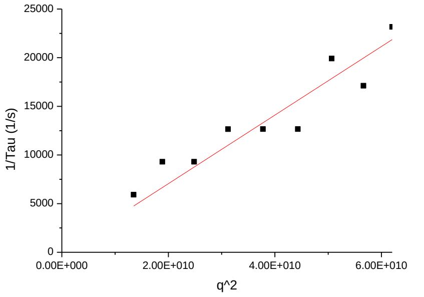
poly(tpy)	THF	0.28	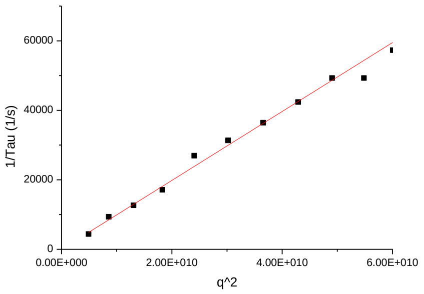
	Toluene	0.16	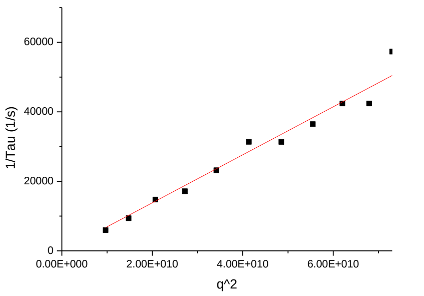

(Fc)-*co*-(tpy)	THF	0.28	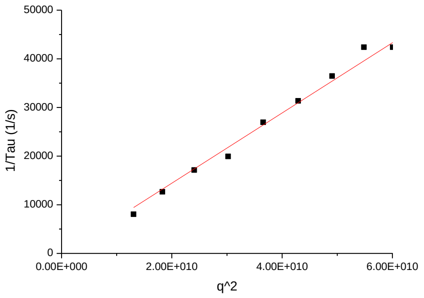
(Fc)-*bl*-(tpy)	THF	0.23	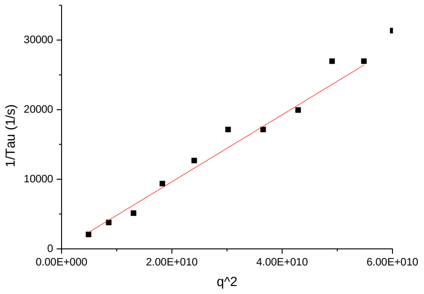

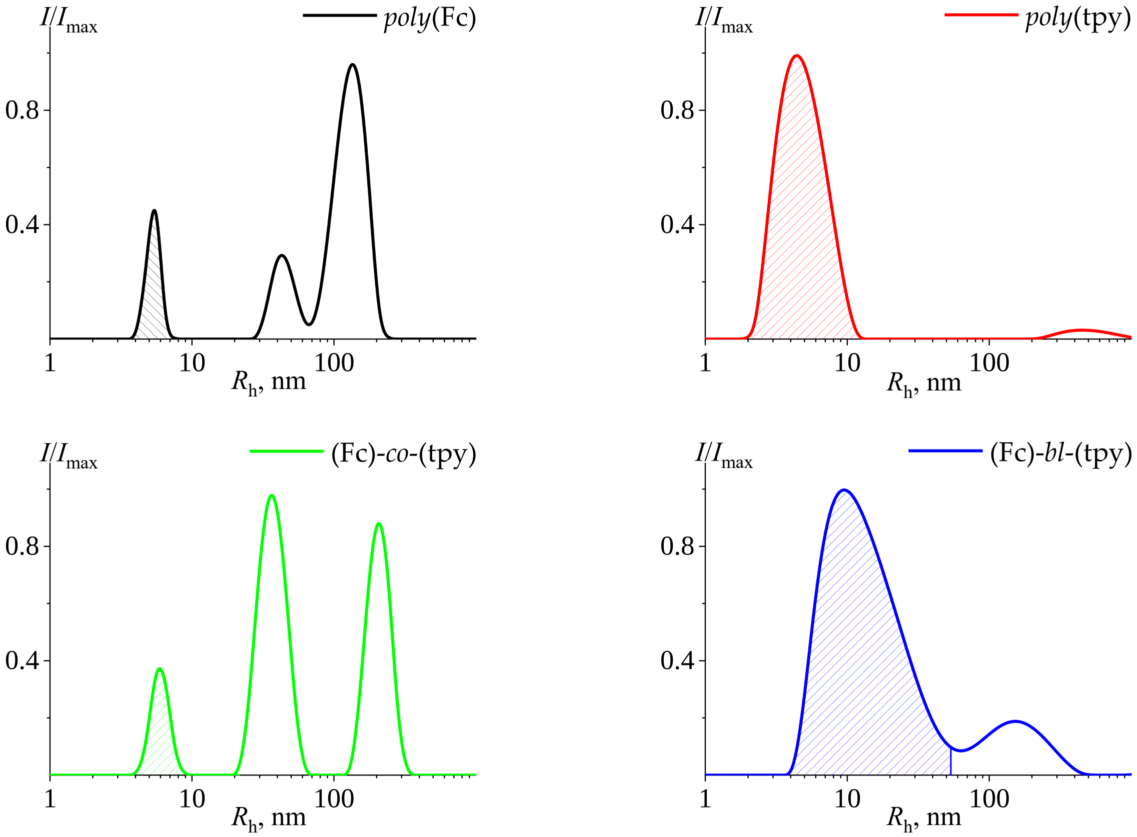

Figure S9. The normalized scattered light intensity distributions I/I_{max} over hydrodynamic radii R_h for the studied polymers and copolymers in THF solutions. All data were obtained at 20 °C. The diffusion coefficients at infinite dilution limit D_0 are presented in Table 3.

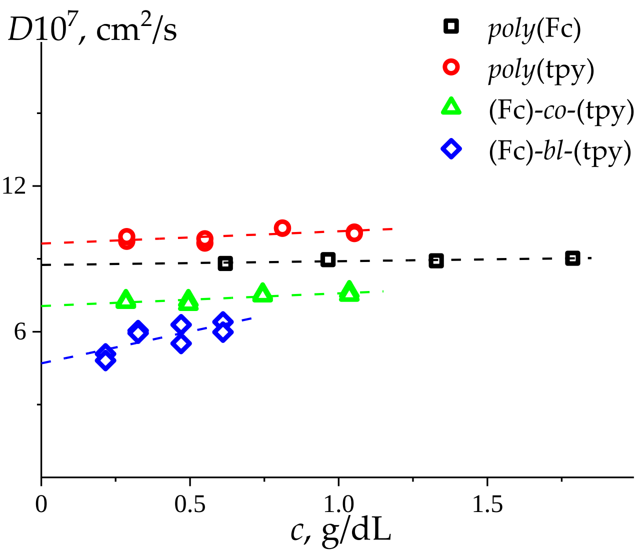

Figure S10. The concentration dependences of diffusion coefficients obtained for THF solutions of studied polymers and copolymers. All data were obtained at 20 °C.

References

1. Dynamic Viscosity of Tetrahydrofuran. Available online: http://www.ddbst.com/en/EED/PCP/VIS_C159.php (accessed on Apr. 6, 2022).
2. Podolyanko, V.A.; Volkova N.D.; Svyatskaya, T.N.; Kostitsyna, O.V. Untersuchung der intermolekularen Wechselwirkung im System Diethylenglykol-Tetrahydrofuran. *Vestn. Khark. Univ.* **1977**, 32-38.
3. Gill, D.S.; Singh, J.; Ludwig, R.; Zeidler, M.D. Nuclear magnetic resonance relaxation, permittivity, viscosity and ultrasonic velocity measurements in binary mixtures of methanol and tetrahydrofuran. *J. Chem. Soc., Faraday Trans.* **1993**, *89*, 3955-3958, https://doi.org/10.1039/ft9938903955.
4. Wencel, A.; Czerepko, K. Refractive Indices, Densities and Viscosities of the Tetrahydrofuran-Water Mixtures Taken at Three Temperatures. *Pol. J. Chem.* **1991**, *65* 1809–1823.
5. Aminabhavi, T.M.; Gopalakrishna, B. Density, Viscosity, Refractive Index, and Speed of Sound in Aqueous Mixtures of N,N-Dimethylformamide, Dimethyl Sulfoxide, N,N-Dimethylacetamide, Acetonitrile, Ethylene Glycol, Diethylene Glycol, 1,4-Dioxane, Tetrahydrofuran, 2-Methoxyethanol, and 2-Ethoxyethanol at 298.15 K. *J. Chem. Eng. Data* **1995**, *40*, 856-861, https://doi.org/10.1021/je00020a026.
6. Ponomarenko, S.M.; Mushtakova, S.P.; Demakhin, A.G.; Faifel', B.L.; Kalmanovich, D.G. Physicochemical Properties and Electronic Structure of some Aprotic Solvents. *Zh. Obshch. Khim.* **1995**, *65*, 190–198.
7. Ramkumar, D.H.S.; Kudchadker, A.P. Mixture properties of the water + γ-butyrolactone + tetrahydrofuran system. Part 2. Viscosities and surface tensions of γ-butyrolactone + water at 303.15-343.15 K and γ-butyrolactone + tetrahydrofuran at 278.15-298.15 K. *J. Chem. Eng. Data* **1989**, *34*, 463-465, https://doi.org/10.1021/je00058a027.
8. Gubarev, A.S.; Lezov, A.A.; Senchukova, A.S.; Vlasov, P.S.; Serkova, E.S.; Kuchkina, N.V.; Shifrina, Z.B.; Tsvetkov, N.V. Diels–Alder Hyperbranched Pyridylphenylene Polymer Fractions as Alternatives to Dendrimers. *Macromolecules* **2019**, *52*, 1882–1891, https://doi.org/10.1021/acs.macromol.8b02388.
